# Supplementary material for: Generation of the Human Pluripotent Stem-Cell-Derived Astrocyte Model with Forebrain Identity
Source: Brain Sci. 2021 Feb 9;11(2):209. doi: 10.3390/brainsci11020209 (PMC7914711; doi:10.3390/brainsci11020209)
Supplement: Supplementary file 1 [file brainsci-11-00209-s001.pdf]

Table S1. Primers used for qPCR analysis.

Gene	Forward primer (5′-3′)	Reverse primer (5′-3′)
GAPDH	AACGACCCCTTCATTGAC	TCCACGACATACTCAGCAC
PAX6	CCTCATTTCCCGCTCTGGTT	CAGATTCCTATGCTGATTGGTGATG
TBR2	CTCCATCTCCCACGGATTCTC	TTCGCTCTGTTGGGGTGAAA
MAP2	GTGCCGAGTGAGAAGAAGGT	AACAATTTGTACCTGCCCCC
GFAP	ACCTGCAGATTCGAGAAACCAG	GGTCCTGCCTCACATCACATC
S100β	GGTGAGACAAGGAAGAGGATGTCTG	ACATTCGCCGTCTCCATCATT
GLAST	ATTCCAGCAGGGAGTCCGTA	TCCAAGGATTGTACCCACAATGA
